# Plantar Exteroceptive Inefficiency causes an asynergic use of plantar and visual afferents for postural control: Best means of remediation

**DOI:** 10.1002/brb3.658

**Published:** 2017-05-01

**Authors:** Arnaud Foisy, Zoï Kapoula

**Affiliations:** ^1^ IRIS Team, Physiopathologie de la Vision et Motricité BinoculaireFR3636 Neurosciences CNRS, University Paris DescartesParisFrance

**Keywords:** feet cutaneous afferents, interindividual differences, Plantar Quotient, Romberg Quotient, thin plantar insert, visual‐podal conflict

## Abstract

**Introduction:**

Some subjects have difficulty to integrate both visual and plantar inputs, showing at the same time a “postural blindness” and a Plantar Exteroceptive Inefficiency (PEI). The former corresponds to a better stability eyes closed (EC) than eyes open (EO), while the latter is defined as a better stability on foam than on firm ground. Clinical studies reported that a manipulation of either plantar or visual input could affect the weight of both cues in postural control, suggesting interdependence in their use. The purpose of the experiment is to characterize the PEI phenomenon better and see if such synergy can be objectified.

**Methods:**

We recruited 48 subjects (25 ± 3.3 years) and assessed their balance with a force platform, EO, EC, at 40 or 200 cm, on firm ground, Dépron^®^ foam, Dynachoc^®^ foam, or on a 3 mm‐thick Anterior Bar AB^®^. We assessed their sensorial preferences through their PQ and RQ.

**Results:**

The main results are that there normally exists a synergy in the use of plantar and visual afferents, but only at 40 cm and in the absence of PEI.

**Conclusions:**

Plantar Exteroceptive Inefficiency interferes with the role of vision in postural control, its effects are distance specific, are better revealed by Dépron^®^ foam and the AB^®^ improves posture but does not solve visual‐podal asynergy. These results also have clinical interests as they indicate the best way in terms of distance and choice of foam to diagnostic PEI. Finally, they suggest restricting the use of the AB^®^, commonly employed. These findings can be useful for clinicians concerned with foot, eye, and posture.

## 1 Introduction

The control of upright posture depends on the correct integration and processing of peripheral afferents (vestibular, visual, and somaesthetic) by the central nervous system (CNS; Ruhe, Fejer, & Walker 2010). Among young and healthy subjects interindividual differences exist, at sensorimotor as well as at perceptual level; some people considerably use their visual afferents for spatial perception and balance, while others do not (Crémieux & Mesure, 1994; Ehrenfried, Guerraz, Thilo, Yardley, & Gresty, 2003; Isableu, Fourre, Vuillerme, Giraudet, & Amorim, 2011; Isableu et al., 2010; Lacour et al., 1997). Normally, healthy subjects are twice as stable with eyes open (EO) as with eyes closed (EC; Le & Kapoula, 2007). However, some subjects show a better stability with EC than EO, which finds expression in a Romberg Quotient below 100 (RQ = *S*_eyes closed_/*S*_eyes open_ × 100, where *S* stands for the Surface of the excursions of the Center of Pressure (CoP)—Severac, Bessou, & Pages, 1994; Van Parys & Nijokitkjien, 1976). Marucchi and Gagey (1987) named that paradoxical situation “postural blindness”.

Likewise, in a healthy population, some subjects rely more on their somatosensory afferents than others (Isableu & Vuillerme, 2006; Kluzik, Horak, & Peterka, 2005; Streepey, Kenyon, & Keshner, 2007). A minority of them are even more stable on foam than on firm ground; this was first reported by Dujols (1991) and later confirmed by other work (Foisy & Kapoula, 2016; Isableu & Vuillerme, 2006; Patel, Fransson, Lush, Gomez, 2008; Yi & Park, 2009). This unusual situation is attributed to a Plantar Exteroceptive Inefficiency (PEI), which is revealed by a Plantar Quotient below 100 (PQ = *S*_foam_/*S*_firm ground_ × 100; Foisy & Kapoula, 2016). PEI is a latent plantar somaesthetic dysfunction (Foisy & Kapoula, 2016; Janin, 2009) due to an increase in pressure beneath certain plantar zones (such as the first metatarsal head) resulting in an increase in the frequency discharge of the sole receptors (Ribot‐Ciscar, Vedel, & Roll, 1989; Vedel & Roll, 1982). The latter constitutes a “noise” hindering the integration of the increased plantar afferents (Weerakkody, Percival, Canny, Morgan, & Proske, 2003). The interposition of foam between the ground and the subjects’ feet smoothes plantar pressure distribution (Chiang & Wu, 1997; Wu & Chiang, 1997) and decreases the plantar signal (Yi & Park, 2009), which normally makes people more unstable. In contrast, in case of PEI, the mitigation of plantar pressure peaks and noisy signal results in a better stability than on firm ground (see Foisy & Kapoula, 2016).

Moreover, clinical observations suggest that some people suffer both from PEI and postural blindness, but the latter is present only when they stand on firm ground (Dujols, 1991; Weber & Gagey, 1998). It suggests interdependence in the use of eye and feet afferents in the control of posture; when the effects of the PEI are reduced by foam interposition, the subjects are able to integrate their visual afferents again, as shown by the increase in their RQ. Dujols (1991) only reported a few clinical cases but a previous experiment of our team brought first evidence of the influence of PEI on oculomotor control (Foisy & Kapoula, 2016). Indeed, we showed that in addition to its postural consequences, the PEI entirely suppresses the influence of thin plantar inserts on oculomotor control.

Postural stability and use of visual afferents in postural control also depends on the distance of the visual target. Le and Kapoula (2007) showed that the RQ of young and old subjects was close to 200 at 20 cm and 40 cm, but dropped to 100 at 90 cm and beyond (200 and 350 cm). Knowing that at near distance the vergence angle is greater than at far distance, thus increasing the proprioceptive signals of the extra‐ocular muscles; the authors concluded that at near distance, the CNS uses vision coupled with oculomotor convergence signals, leading to high RQ. They suggested that at intermediate and far distances, the CNS would use mostly internal signals (somatosensory). However, the latter suggestion was a hypothesis that they did not actually assess. In this experiment, the measurement of both the RQ and the PQ at near and far distances allows us to test this hypothesis. Hence, the goal of this study is to characterize subjects with and without PEI for all these conditions and thus see if a visual‐podal synergy can be objectified.

Furthermore, in a clinical study, Janin (2002) suggested that thin inserts set just behind the metatarsal heads (Anterior Bars [ABs^®^ ‐ filed by Sylvie and Philippe Villeneuve, INPI N° 938 925 to 938 841]) were more efficient than foam in reducing pressure peaks beneath the heads. Those excessive pressures being considered to cause PEI (Janin, 2009), a supplementary goal which has a major clinical interest, is to test whether slightly different foam surfaces and ABs^®^ (commonly used in clinical practice ‐ Bourdiol 1986, Villeneuve 1990) have the same effects on PQ, RQ, and postural performances.

These questions are important to answer because PEI is frequently encountered in clinical practice and better understanding of its pathophysiology could lead to better care of the involved patients.

## 2 Material and Methods

### 2.1 Ethics statement

The investigation adhered to the principles of the Declaration of Helsinki and was approved by the “Conseil d'Evaluation Éthique pour les Recherches en Santé” University Paris Descartes, No. IRB: 20153300001072. The subjects gave informed written consent after the nature of the procedure was explained.

### 2.2 Subjects

Forty‐height healthy young subjects took part in the study. They were recruited from paramedical schools: 21 males and 27 females, mean age 25 ± 3.3 years, mean height 170.1 ± 8.6 cm, mean body weight 63.7 ± 10.5 kg. Their characteristics are summarized in Table S1.

None of them were taking medication and all of them were asymptomatic. All subjects were emmetropic and wore no glasses. Their visual acuity at close distance was examined by means of Parinaud's reading test. The results were all normal (47 subjects scored 2, one of them scored 3). Binocular visual function was also assessed with the stereoacuity TNO test and all values were normal, that is, 60″ of arc or lower. We also measured the Near Convergence Point, which was 5.06 ± 1.82 cm, and the amplitude of accommodation with the push‐up method (we did a mean of three measures for both tests; Duane, 1912; Rutstein, Fuhr, & Swiatocha, 1993). The subjects had a mean of 9.37 dioptres (±1.85), which is within Duane's normative data (9.5 ± 2 dioptres; Duane, 1912). The *t* test did not show any statistical difference relative to that theoretical physiologic value (*p *=* *.616).

### 2.3 Postural performances assessment

We assessed the postural performances of our subjects in quiet stance with a force platform consisting of two clogs (produced by TechnoConcept, Céreste, France and using the Standards of the Association Française de Posturologie). The position of the clogs was standardized: feet placed side by side, forming a 30° angle with heels separated by 4 cm. Each clog holds two strain gauges (one beneath the metatarsal heads, one beneath the heel) which are force—electric tension transducers. The height and weight of the subjects were factored into the calculations of the CoP displacements. Following the recommendation of Pinsault and Vuillerme (2009), the CoP displacements were recorded over three periods of 25.6 s. The equipment contained an Analogue—Digital converter of 16 bits and the sampling frequency of the CoP was 40 Hz. We analyzed the classical postural parameters: the Surface area of CoP excursions and the Variance of Speed of CoP. The Surface area of the CoP represents 90% of the instantaneous positions of the CoP included within the confidence ellipse, eliminating the extreme points (Ruhe, Fejer, & Walker, 2013).

The subjects were asked to stand still, barefoot, on the force plate, and stare at a target in front of their eyes. There were four plantar stimulation conditions: (1) on firm ground (control condition); (2) on Dépron^®^ foam (6 mm‐thick, shore rating of 20A, density of 33 kg/m^3^, commercialized by Chaleurosol SARL, France); (3) on Dynachoc^®^ foam (3 mm‐thick, shore rating of 35A, density of 350 kg/m^3^, commercialized by BL3D SAS, France); (4) with an AB^®^ set just backward the metatarsal heads (a 3 mm‐thick plantar insert, shore rating of 60A, density of 250 kg/m^3^). We tested each plantar condition with EO, EC, at close distance (40 cm) and far distance (200 cm), so that there were in total 16 counterbalanced testing conditions.

After a first familiarization trial, the postural performances of each subject were recorded three times for each condition; means of those measures were calculated. In order to avoid a phenomenon of habituation of the sole, cutaneous mechanoreceptors, a 1‐min period of seated rest separated each recording (Pinsault & Vuillerme, 2009).

### 2.4 Sensorial preferences assessment

Thanks to those recordings, we were able to calculate the subjects’ Plantar Quotient. The PQ consists of the ratio between the Surface area of the CoP excursions while the subjects stand on foam and the Surface area while they stand on firm ground: PQ = *S*_foam_/*S*_firm ground_ × 100 (Dujols, 1991). Foam decreases the information arising from the feet (Yi & Park, 2009), normally resulting in a decreased stability (Chiang & Wu, 1997; Isableu & Vuillerme, 2006; Patel, Fransson, Lush, & Gomez, 2008; Patel, Fransson, Lush, Petersen, et al., 2008; Wu & Chiang, 1997), indicated by a PQ > 100. Therefore, the PQ provides information on the weight of plantar cutaneous afferents used in postural control (Isableu et al., 2011; Oie, Kiemel, & Jekka, 2002): the higher it is, the more the subject relies on the information arising from his feet to keep balance. In the literature, thick (several cm) and compliant foam support surfaces are generally used, leading to both biomechanical and sensorial effects (Patel, Fransson, Lush, Gomez, 2008; Patel, Fransson, Lush, Petersen, et al., 2008; Yi & Park, 2009), the latter involving plantar exteroception and proprioception at the same time (Chiang & Wu, 1997; Patel, Fransson, Lush, Gomez, 2008; Patel, Fransson, Lush, Petersen, et al., 2008; Wu & Chiang, 1997). Here, we used thin and firm foam in order to focus the action on plantar cutaneous afferents (following Dujols, 1991; Leporck & Villeneuve, 1996; Foisy & Kapoula, 2016).

Likewise, we calculated the Romberg Quotient (RQ = *S*_eyes closed_/*S*_eyes open_ × 100), on firm ground or foam, at 40 and 200 cm. In the same way, this ratio betrays the influence of visual afferents in postural control.

Previous work showed that the PQ (Foisy & Kapoula, 2016) and the RQ (Brandt, Paulus, & Straube, 1986; Kapoula & Le, 2006; Le & Kapoula, 2007) are valuable tools to account for interindividual differences in the use of somatosensory and visual cues. Shumway‐Cook and Horak (1986) were the first to propose a clinical assessment of the sensory interactions on balance, thanks to eye closure and interposition of foam pads. Later, comparisons of the PQ EO and EC, or of the RQ on firm ground or on foam were used by several authors to assess the weight of those inputs in postural control: Fujimoto et al. (2009) (“foam ratios”), Di Berardino et al. (2009) (“sensory ratios”), or Preszner‐Domjan et al. (2012) among healthy subjects.

### 2.5 Statistical analysis

Statistical analysis was performed using nonparametric tests, that is, Mann–Whitney *U* tests, Kruskal–Wallis, or Friedman's test (procedure of Statsoft/Statistica, release 7.1) since the test of Shapiro–Wilk revealed that some of the distributions were not normal and proved impossible to normalize. Post hoc comparisons were done whenever necessary using the test of Wilcoxon, with *p *<* *.05 considered significant. The magnitudes of the differences were assessed by the effect size (Cohen's *d*).

We applied the Bonferroni‐Holm method correction for multiple testing (Aickin & Gensler, 1996; Holm, 1979), and the corrected *p*‐values are shown in the text.

## 3 Results

### 3.1 Group comparisons

We obtained a mean PQ of 112 ± 39 in the baseline condition (EO on firm ground at 40 cm) and divided our population into two groups: the Normal Plantar Quotient Subjects (NPQS), who showed a normal response, being more stable on firm ground than on foam (PQ > 100: 30 subjects, with a mean PQ of 136 ± 28); and the Plantar Exteroceptive Inefficient Subjects (PEIS) who had a PQ below 100 (that is 18 subjects, with a mean PQ of 73 ± 14). We compared the subjects’ basic characteristics of the two groups; and then we compared their PQ, RQ, and postural performances.

#### 3.1.1 Basic characteristics

Mann–Whitney *U* tests showed that the two groups did not have significantly different ages (*z* = −0.07, *p = *.94), heights (*z* = −1.34, *p = *.18), weights (*z* = −1.12, *p = *.26), stereoacuity (*z* = 0.38, *p = *.70), visual acuity (*z* = −0.38, *p = *.70), amplitude of accommodation (*z* = −1.27, *p = *.21), and Near Convergence Point (*z* = 0.42, *p = *.68).

#### 3.1.2 Plantar Quotient and Romberg Quotient according to distance and to the group

**Figure 1:**
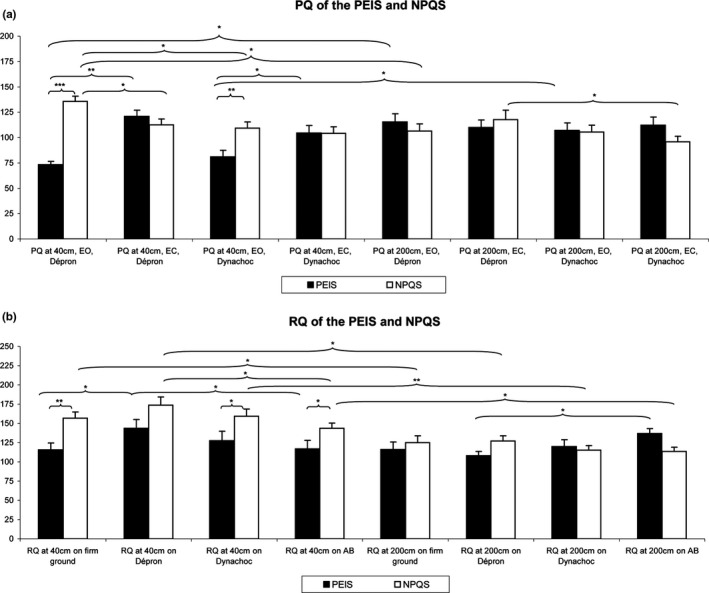
Mean Plantar Quotient (PQ) (a) and Romberg Quotient (RQ) (b) among the Normal Plantar Quotient Subjects (NPQS) and the Plantar Exteroceptive Inefficient Subjects (PEIS). Error bars represent the standard errors. Asterisks indicate significant differences, with *p <* *.05; **p <* *.01, ***p <* *.001

At 40 cm, the PEIS had a lower PQ than the NPQS, EO on Dépron^®^ (*z* = −5.75, *p < *.01, *d *=* *2.85) and on Dynachoc^®^ (*z* = −3.15, *p < *.01, *d *=* *2.85; Figure 1a).

At 40 cm, the PEIS had a lower RQ than the NPQS on firm ground (*z* = −3.26, *p < *.01, *d *=* *0.98), on Dynachoc^®^ (*z* = −2.17, *p = *.03, *d *=* *0.60), and on AB^®^ (*z* = −2.39, *p = *.02, *d *=* *0.61). In contrast, their RQ on Dépron^®^ displayed no significant differences (*z* = −1.75, *p = *.08). At 200 cm, their RQ on firm ground (*z* = −0.34, *p = *.73), on Dynachoc^®^ (*z* = 0.17, *p = *.87), and on Dépron^®^ (*z* = −1.66, *p = *.10) was not significantly different. The RQ of the NPQS on AB^®^ was significantly lower than the one of the PEIS (*z* = −2.72, *p = *.01, *d *=* *0.85; Figure 1b).

The results are summarized in Figure 1 and Table 1.

**Table 1:** Quotients and postural performances

Condition	Plantar Quotient (PQ)		Romberg Quotient (RQ)		Surface area of CoP (mm^2^)		Variance of Speed (mm^2^/s^2^)	
	NPQS	PEIS	NPQS	PEIS	NPQS	PEIS	NPQS	PEIS
Eyes open (EO)								
Firm ground 40 cm			157 ± 8, [140, 173]	116 ± 9, [97, 135]	104 ± 11, [82, 127]	133 ± 13, [105, 160]	24 ± 2, [20, 28]	29 ± 3, [21, 36]
Dépron^®^ 40 cm	136 ± 5, [125, 146]	73 ± 3, [67, 80]	174 ± 10, [153, 195]	143 ± 12, [119, 168]	133 ± 12, [110, 157]	101 ± 13, [74, 129]	26 ± 2, [22, 30]	24 ± 3, [18, 30]
Anterior Bar^®^ 40 cm			143 ± 7, [129, 158]	117 ± 11, [94, 140]	109 ± 11, [87, 132]	106 ± 14, [76, 135]	23 ± 2, [19, 27]	27 ± 3, [20, 30]
Dynachoc^®^ 40 cm	109 ± 6, [97, 122]	81 ± 6, [67, 95]	159 ± 10, [139, 179]	127 ± 12, [101, 154]	103 ± 9, [85, 121]	109 ± 13, [82, 137]	22 ± 2, [17, 27]	26 ± 3, [19, 32]
Firm ground 200 cm			125 ± 9, [107, 143]	116 ± 9, [96, 136]	131 ± 14, [101, 160]	139 ± 17, [102, 176]	29 ± 3, [23, 34]	31 ± 4, [22, 39]
Dépron^®^ 200 cm	107 ± 7, [92, 121]	115 ± 8, [98, 133]	127 ± 7, [113, 141]	108 ± 6, [96, 120]	128 ± 11, [105, 151]	148 ± 16, [115, 181]	28 ± 3, [23, 33]	31 ± 4, [23, 39]
Anterior Bar^®^ 200 cm			113 ± 5, [102, 125]	137 ± 6, [124, 150]	113 ± 9, [93, 132]	105 ± 10, [85, 125]	25 ± 2, [21, 30]	27 ± 3, [21, 34]
Dynachoc^®^ 200 cm	105 ± 7, [92, 119]	107 ± 7, [91, 123]	115 ± 6, [103, 128]	120 ± 9, [101, 139]	122 ± 10, [101, 144]	138 ± 14, [110, 167]	28 ± 3, [22, 33]	29 ± 4, [21, 36]
Eyes closed (EC)								
Firm ground 40 cm					161 ± 17, [126, 196]	168 ± 23, [120, 216]	39 ± 4, [32, 47]	44 ± 5, [33, 55]
Dépron^®^ 40 cm	112 ± 6, [100, 125]	121 ± 6, [108, 134]			178 ± 17, [143, 211]	194 ± 24, [144, 245]	41 ± 4, [34, 48]	50 ± 6, [36, 63]
Anterior Bar^®^ 40 cm					151 ± 15, [120, 181]	154 ± 19, [114, 193]	37 ± 3, [30, 43]	46 ± 6, [34, 58]
Dynachoc^®^ 40 cm	104 ± 7, [91, 118]	105 ± 7, [89, 120]			158 ± 16, [125, 191]	170 ± 22, [124, 216]	38 ± 4, [31, 45]	42 ± 5, [31, 53]
Firm ground 200 cm					154 ± 14, [125, 182]	148 ± 14, [119, 178]	42 ± 4, [34, 50]	40 ± 5, [29, 50]
Dépron^®^ 200 cm	118 ± 9, [99, 137]	110 ± 7, [94, 126]			167 ± 15, [136, 198]	156 ± 14, [125, 186]	41 ± 4, [33, 50]	43 ± 5, [32, 54]
Anterior Bar^®^ 200 cm					132 ± 14, [103, 161]	161 ± 17, [126, 197]	36 ± 3, [29, 42]	44 ± 5, [32, 55]
Dynachoc^®^ 200 cm	96 ± 5, [85, 107]	112 ± 8, [95, 129]			145 ± 15, [115, 175]	167 ± 20, [125, 209]	38 ± 4, [30, 46]	41 ± 6, [29, 53]

Means, standard errors, and 95% Confidence Intervals of quotients and postural parameters for each condition among the Normal Plantar Quotient Subjects (NPQS) and the Plantar Exteroceptive Inefficient Subjects (PEIS).

#### 3.1.3 Postural performances according to the group

In relation to their postural performances, the only significant difference between the two groups was their Surface area on firm ground at 40 cm, EO: the PEIS had a higher Surface than the NPQS (*z* = 1.99, *p = *.05, *d *=* *0.50).

### 3.2 Interaction in the use of plantar cutaneous and visual afferents and effects of foam surfaces and Anterior Bar

We compared the PQ on all the conditions (i.e., on Dépron^®^, on Dynachoc^®^, EO and EC, at 40 cm and at 200 cm) independently for each subgroup (PEIS, NPQS) since we could not resort to parametric ANOVAs with independent factors.

#### 3.2.1 Plantar Quotient comparisons

##### For the Normal Plantar Quotient Subjects

For the NPQS, the Friedman's test showed a main effect on the PQ ($\chi_{7,30}^{2}$ = 24.70, *p *<* *.01). The test of Wilcoxon showed that, at 40 cm, their PQ was significantly lower EC than EO on Dépron^®^ (*z* = 3.14, *p *=* *.02, *d *=* *0.80). Their PQ was also significantly higher at 40 cm than at 200 cm EO on Dépron^®^ (*z* = 3.18, *p *=* *.02, *d *=* *0.85). As concerns the effects of foam surfaces on the PQ, the test showed that at 40 cm, the PQ on Dynachoc^®^ was significantly lower than the PQ on Dépron^®^ EO (*z* = 3.20, *p *=* *.02, *d *=* *0.88). At 200 cm, the PQ on Dynachoc^®^ was significantly lower than the PQ on Dépron^®^ EC (*z* = 2.68, *p *=* *.04, *d *=* *0.55; Figure 1a).

##### For the Plantar Exteroceptive Inefficient Subjects

For the PEIS, there was a main effect on the PQ ($\chi_{7,18}^{2}$ = 38.26, *p *<* *.01). Contrary to the NPQS, at 40 cm, their PQ was significantly *higher* EC than EO on Dépron^®^ (*z* = 3.68, *p *<* *.01, *d *=* *2.30) and on Dynachoc^®^ (*z* = 2.55, *p *=* *.04, *d *=* *0.83). Their PQ was also significantly *lower* at 40 cm than at 200 cm EO on Dépron^®^ (*z* = 3.11, *p *=* *.02, *d *=* *1.62) and on Dynachoc^®^ (*z* = 2.20, *p = *.03, *d *=* *0.89). There was no difference in relation to the effects of foam surfaces on the PQ among the PEIS (Figure 1a).

#### 3.2.2 Romberg Quotient comparisons

Likewise, we compared the RQ on all the conditions (i.e., on firm ground, on Dépron^®^, and on Dynachoc^®^, at 40 cm and at 200 cm) independently for each subgroup.

##### For the Normal Plantar Quotient Subjects

For the NPQS, there was a main effect on the RQ ($\chi_{7,30}^{2}$ = 46.46, *p *<* *.01). At 40 cm, the RQ on Dépron^®^ was significantly higher than the RQ on AB^®^ (*z* = 2.56, *p *=* *.04, *d *=* *0.64). As concerns the effects of distance on the RQ, the test showed that at 200 cm, the RQ was significantly lower than at 40 cm on firm ground (*z* = 2.58, *p *=* *.05, *d *=* *0.68), on Dépron^®^ (*z* = 3.18, *p *=* *.02, *d *=* *0.98), on Dynachoc^®^ (*z* = 3.48, *p *=* *.01, *d *=* *1.00), and on AB^®^ (*z* = 2.93, *p *=* *.05, *d *=* *0.88; Figure 1b).

##### For the Plantar Exteroceptive Inefficient Subjects

For the PEIS, there was a borderline main effect on the RQ ($\chi_{7,18}^{2}$ = 13.33, *p *=* *.06). At 40 cm, the RQ on Dépron^®^ was significantly higher than the RQ on firm ground (*z* = 2.98, *p *=* *.05, *d *=* *0.61), higher than the RQ on AB^®^ (*z* = 3.03, *p *=* *.04, *d *=* *0.54), and there was a borderline difference between the RQ on Dépron^®^ and on Dynachoc^®^, the former being higher (*z* = 1.81, *p *=* *.07, *d *=* *0.31). At 200 cm, the RQ on Dépron^®^ was significantly lower than the RQ on AB^®^ (*z* = 2.81, *p *=* *.05, *d *=* *1.14). There was no significant effect of distance on the RQ of the PEIS (Figure 1b).

#### 3.2.3 Postural performances comparisons

**Figure 2:**
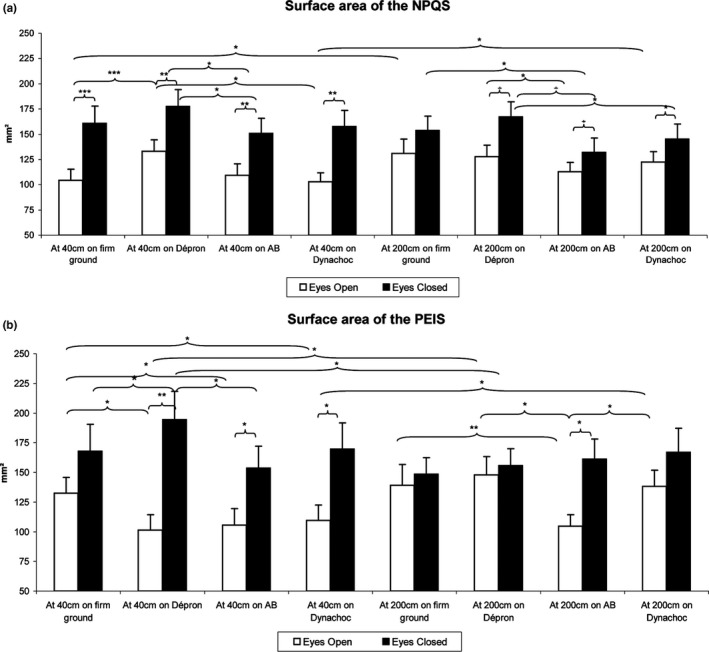
Mean Surface area for each testing condition among the Normal Plantar Quotient Subjects (NPQS) (a) and the Plantar Exteroceptive Inefficient Subjects (PEIS) (b). Error bars represent the standard errors. Asterisks indicate significant differences, with *p < .05 **p <* *.01, ***p <* *.001

**Figure 3:**
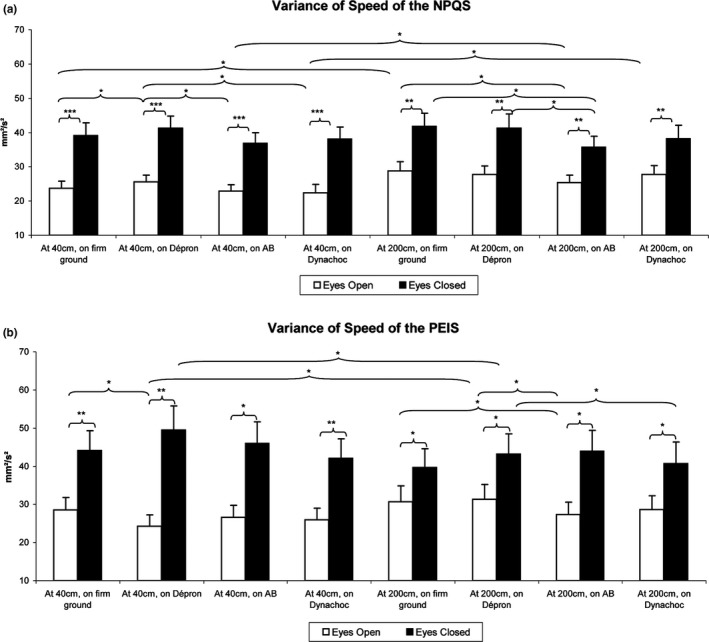
Mean Variance of Speed among the Normal Plantar Quotient Subjects (NPQS) (a) and the Plantar Exteroceptive Inefficient Subjects (PEIS) (b). Error bars represent the standard errors. Asterisks indicate significant differences, with *p <* *.05; **p <* *.01, ***p <* *.001

Finally, we compared the postural performances among each subgroup. The results are summarized in Figures 2 and 3 and Table 1.

##### For the Normal Plantar Quotient Subjects

For the NPQS, there was a main effect on the Surface area ($\chi_{15,30}^{2}$ = 127.69, *p *<* *.01). The test of Wilcoxon showed effects of the ground condition: at 40 cm EO, the Surface area was smaller on firm ground than on Dépron^®^ (*z* = 4.78, *p *<* *.01, *d *=* *0.47), on AB^®^ compared to Dépron^®^ (*z* = 3.12, *p *=* *.05, *d *=* *0.37), and on AB^®^ compared to Dynachoc^®^ (*z* = 3.18, *p *=* *.05, *d *=* *0.11). At 40 cm EC, the Surface area was smaller on AB^®^ than on Dépron^®^ (*z* = 2.77, *p *=* *.05, *d *=* *0.56). At 200 cm EO, the Surface area was smaller with AB^®^ compared to Dépron^®^ (*z* = 1.98, *p *=* *.05, *d *=* *0.26). At 200 cm EC, it was smaller with AB^®^ compared to firm ground (*z* = 2.31, *p *=* *.02, *d *=* *0.29), and Dépron^®^ (*z* = 3.36, *p *=* *.03, *d *=* *0.44). The Surface area was also smaller on Dynachoc^®^ than on Dépron^®^ (*z* = 2.09, *p *=* *.04, *d *=* *0.27). There also was an effect of distance: the Surface area was smaller at 40 cm than at 200 cm EO on firm ground (*z* = 2.70, *p *=* *.05, *d *=* *0.38) and Dynachoc^®^ (*z* = 2.29, *p *=* *.02, *d *=* *0.36). As concerns the effects of eye closure, it was correlated with a significant increase in the Surface area at 40 cm on firm ground (*z* = 4.56, *p *<* *.01, *d *=* *0.73), Dépron^®^ (*z* = 3.90, *p *<* *.01, *d *=* *0.57), AB^®^ (*z* = 3.69, *p *=* *.01, *d *=* *0.54), and Dynachoc^®^ (*z* = 4.14, *p *<* *.01, *d *=* *0.78). At 200 cm, there was also a borderline difference between the Surface area EC and EO on firm ground (the Surface EC being higher) (*z* = 1.82, *p* = .07, *d* = 0.30), and a significant higher Surface area EC than EO on Dépron^®^ (*z* = 3.55, *p *=* *.02, *d *=* *0.53), AB^®^ (*z* = 2.05, *p *=* *.04, *d *=* *0.29), and Dynachoc^®^ (*z* = 2.46, *p *=* *.04, *d *=* *0.33; Figure 2a).

Another Friedman's test showed a main effect on the Variance of Speed ($\chi_{15,30}^{2}$ = 206.43, *p *<* *.01). At 40 cm EO, the Variance of Speed was lower on firm ground (*z* = 2.06, *p *=* *.04, *d *=* *0.18), on AB^®^ (*z* = 2.72, *p *=* *.05, *d *=* *0.29), and on Dynachoc^®^ (*z* = 2.27, *p *=* *.05, *d *=* *0.32) than on Dépron^®^. At 200 cm EO, the Variance of Speed was lower with AB^®^ compared to firm ground (*z* = 2.21, *p *=* *.03, *d *=* *0.29). At 200 cm EC, it was lower with AB^®^ compared to firm ground (*z* = 2.48, *p *=* *.04, *d *=* *0.31) and to Dépron^®^ (*z* = 2.40, *p *=* *.05, *d *=* *0.24). There also was an effect of distance: the Variance of Speed was lower at 40 cm than at 200 cm EO on firm ground (*z* = 3.25, *p *=* *.05, *d *=* *0.38), AB^®^ (*z* = 2.01, *p *=* *.04, *d *=* *0.18), and Dynachoc^®^ (*z* = 3.53, *p *=* *.02, *d *=* *0.07). The effects of eye closure were correlated with a significant increase in the Variance of Speed at 40 cm on firm ground (*z* = 4.58, *p *<* *.01, *d *=* *0.93), Dépron^®^ (*z* = 4.72, *p *<* *.01, *d *=* *0.97), AB^®^ (*z* = 4.78, *p *<* *.01, *d *=* *1.00), and Dynachoc^®^ (*z* = 4.51, *p *<* *.01, *d *=* *0.96). At 200 cm, the Variance of Speed EC was also significantly higher than EO on firm ground (*z* = 4.12, *p *<* *.01, *d *=* *0.71), Dépron^®^ (*z* = 3.92, *p *<* *.01, *d *=* *0.68), AB^®^ (*z* = 3.92, *p *<* *.01, *d *=* *0.72), and Dynachoc^®^ (*z* = 3.92, *p *<* *.01, *d *=* *0.56; Figure 3a).

##### For the Plantar Exteroceptive Inefficient Subjects

For the PEIS, there was a main effect on the Surface area ($\chi_{15,18}^{2}$ = 103.81, *p *<* *.01). At 40 cm EO, the Surface area was *higher* on firm ground than on Dépron^®^ (*z* = 3.64, *p *=* *.01, *d *=* *0.57), AB^®^ (*z* = 3.16, *p *=* *.05, *d *=* *0.47), and Dynachoc^®^ (*z* = 3.03, *p *=* *.05, *d *=* *0.43). At 40 cm EC, the Surface area was smaller on firm ground (*z* = 2.24, *p *=* *.02, *d *=* *0.26), and on BA (*z* = 2.98, *p *=* *.05, *d *=* *0.44) than on Dépron^®^. At 200 cm EO, it was smaller with AB^®^ compared to firm ground (*z* = 2.50, *p *=* *.05, *d *=* *0.57), Dépron^®^ (*z* = 3.55, *p *=* *.02, *d *=* *0.78), and Dynachoc^®^ (*z* = 3.16, *p *=* *.05, *d *=* *0.66). There also was an effect of distance: the Surface area was smaller at 40 cm than at 200 cm EO on Dépron^®^ (*z* = 2.77, *p *=* *.05, *d *=* *0.77) and Dynachoc^®^ (*z* = 2.29, *p *=* *.04, *d *=* *0.51). As concerns the effects of eye closure, it was correlated with a significant increase in the Surface area at 40 cm on Dépron^®^ (*z* = 3.68, *p *=* *.01, *d *=* *0.94), AB^®^ (*z* = 3.46, *p *=* *.02, *d *=* *0.69), and Dynachoc^®^ (*z* = 3.55, *p *=* *.02, *d *=* *0.80). At 200 cm, the Surface area EC was also significantly higher on AB^®^ (*z* = 3.46, *p *=* *.02, *d *=* *0.97), and there was a borderline difference between the Surface area EC than EO on Dynachoc^®^, the Surface EC being higher than EO (*z* = 1.81, *p *=* *.07, *d *=* *0.40; Figure 2b).

There also was a main effect on the Variance of Speed ($\chi_{15,18}^{2}$ = 148.59, *p *<* *.01). At 40 cm EO, the Variance of Speed was *higher* on firm ground than on Dépron^®^ (*z* = 2.59, *p *=* *.05, *d *=* *0.38). At 40 cm EC, the Variance of Speed was lower on Dynachoc^®^ than on Dépron^®^ (*z* = 2.37, *p *=* *.04, *d *=* *0.32). At 200 cm EO, it was lower with AB^®^ compared to firm ground (*z* = 2.01, *p *=* *.04, *d *=* *0.25) and to Dépron^®^ (*z* = 2.55, *p *=* *.04, *d *=* *0.27). There was an effect of distance: the Variance of Speed was lower at 40 cm than at 200 cm EO on Dépron^®^ (*z* = 3.16, *p *=* *.05, *d *=* *0.49). As concerns the effects of eye closure, it was correlated with a significant increase in the Variance of Speed at 40 cm on firm ground (*z* = 3.68, *p *=* *.01, *d *=* *0.81), Dépron^®^ (*z* = 3.72, *p *=* *.01, *d *=* *1.24), AB^®^ (*z* = 3.64, *p *=* *.01, *d *=* *0.97), and Dynachoc^®^ (*z* = 3.68, *p *=* *.01, *d *=* *0.89). At 200 cm, the Variance of Speed EC was also significantly higher than EO on firm ground (*z* = 3.29, *p *=* *.04, *d *=* *0.46), Dépron^®^ (*z* = 2.94, *p *=* *.05, *d *=* *0.61), AB^®^ (*z* = 3.55, *p *=* *.02, *d *=* *0.89), and Dynachoc^®^ (*z* = 2.46, *p *=* *.04, *d *=* *0.60; Figure 3b).

## 4 Discussion

The main result of this experiment is that there normally exists a synergy in the use of plantar and visual afferents, but only at close distance and in the absence of PEI. The PEIS have a lower Romberg Quotient than the NPQS except on Dépron^®^ foam, thus objectifying a visual‐podal asynergy. The results show that the effects of PEI are visual‐context specific, are better revealed by Dépron^®^ foam and that the AB^®^ improves posture but does not solve visual‐podal asynergy. Hence, the diagnosis of PEI requires careful examination taking into account all of those parameters.

### 4.1 Group comparisons

The PEIS and NPQS are not significantly different regarding their age, anthropometric characteristics, or visual and oculomotor performances. The only significant differences between them are their PQ, RQ, and Surface area. Both, their PQ on Dépron^®^ and on Dynachoc^®^ are different, suggesting that both foams are able to detect PEI.

Regarding their postural performances, the Surface area is higher for the PEIS only on firm ground, EO, at 40 cm, showing that they are less stable than the NPQS in this condition. Furthermore, on Dépron^®^ (EO, at 40 cm), the PEIS show a tendency to be more stable than the NPQS. These results confirm and complement the literature: a previous experiment showed that such differences existed at 90 cm (Foisy & Kapoula, 2016).

Concerning their RQ, the PEIS have a significantly lower RQ at 40 cm than the NPQS on each ground condition, except on Dépron^®^ (see explanation below and Figure 5). This lower RQ is due to a greater Surface area EO (not to a smaller Surface area EC). It demonstrates that the PEIS have difficulties in properly integrating their visual afferents, except when their PEI is suppressed by the interposition of Dépron^®^. This result suggests that their visual afferents cannot be properly used by the CNS for postural control because of the presence of the PEI. In contrast, at a greater distance (200 cm), this phenomenon is not observed anymore, the NPQS displaying even a lower RQ with the ABs^®^ suggesting that this plantar stimulation hinders the integration of their visual afferents at far distance.

### 4.2 Interaction in the use of plantar cutaneous and visual afferents

There are differences in opposite ways between PEIS and NPQS in relation to the influence of eye closure and distance on PQ. Eye closure (only at 40 cm) and distance induce a decrease in the PQ among the NPQS, and conversely an increase among the PEIS. In other words, the NPQS make greater use of their feet and eye cues altogether at close distance than at far, whereas this synergy is missing among the PEIS. As concerns the RQ, only the PEIS’ RQ is affected by the ground condition, rising with Dépron^®^ at 40 cm. Furthermore, the decrease in the RQ with distance is present only among the NPQS; the PEIS’ RQ being already low at close distance. It confirms and complements the results of Le and Kapoula (2007), which showed that the RQ decreases toward 100 when the distance of the visual target increases.

**Figure 4:**
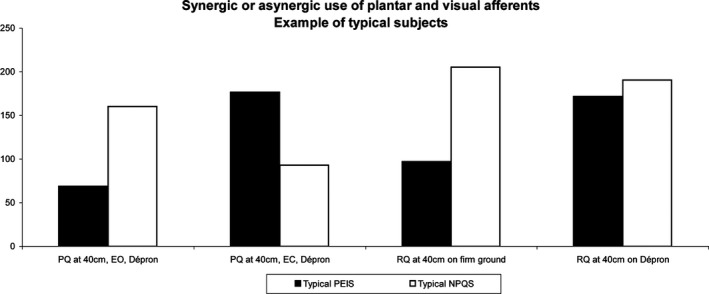
Synergic or asynergic use of plantar and visual afferents. Example of typical subjects. At close distance, the NPQS shows a synergic use of plantar and visual afferents: both his Plantar Quotient (PQ) and Romberg Quotient (RQ) are high (above 100), but the PQ decreases eyes closed. In contrast, the Plantar Exteroceptive Inefficient Subjects (PEIS) shows an asynergic use of plantar and visual afferents; he is unable to properly use both his plantar and visual afferents simultaneously, but when one source of information is diminished (foam interposition or eye closure), the use of the other input rises, as shown by the increase in the corresponding quotient

Taken together, these results (PQ and RQ) show that the quality of integration of plantar cutaneous afferents affects postural control and affects the quality of integration of visual afferents (PEIS have a lower RQ on firm ground), but only at close distance. In other words, PEI can interfere with the role of vision in postural control at near. These observations suggest that, physiologically, at close distance there is a synergy in the use of plantar and visual afferents in order to ensure postural control, but such is not the case at far distance and among the PEIS, who show a visual‐podal asynergy. The effects of PEI only appear at close distance and the visual‐podal asynergy can be suppressed either by foam (Dépron^®^) interposition between the feet and the ground, or by eye closure. Hence, it suggests that these effects are not the consequence of a physical lesion but rather of the difficulty in using the plantar and visual/oculomotor inputs in synergy. This is in line with previous propositions considering that PEI is due to a non‐noxious dysfunction of the sole receptors consisting in a latent increase in their frequency discharge which prevents the CNS from correctly processing and using feet somatosensory afferents (Foisy & Kapoula, 2016; Janin, 2009). Figure 4 gives an example of a typical PEIS and NPQS to illustrate the visual‐podal synergy or asynergy.

We put forward that somatosensory cues are normally used both for balance and vergence control, common zones of the CNS able to exchange information arising from feet and eye inputs (Foisy, Gaertner, Matheron, & Kapoula, 2015; Foisy & Kapoula, 2016). The existence of such common integration zones had already been suggested by other authors: Hollands, Marple‐Horvat, Henkes, and Rowan (1995), Hollands, Ziarva, and Bronstein (2004) proposed the cerebellum or the superior colliculus; and more recently, several studies have shown that cross‐sensory interactions are common in primary cortical areas, most especially in early visual cortex (V1) for visual‐tactile interactions (Lunghi & Alais, 2015).

**Figure 5:**
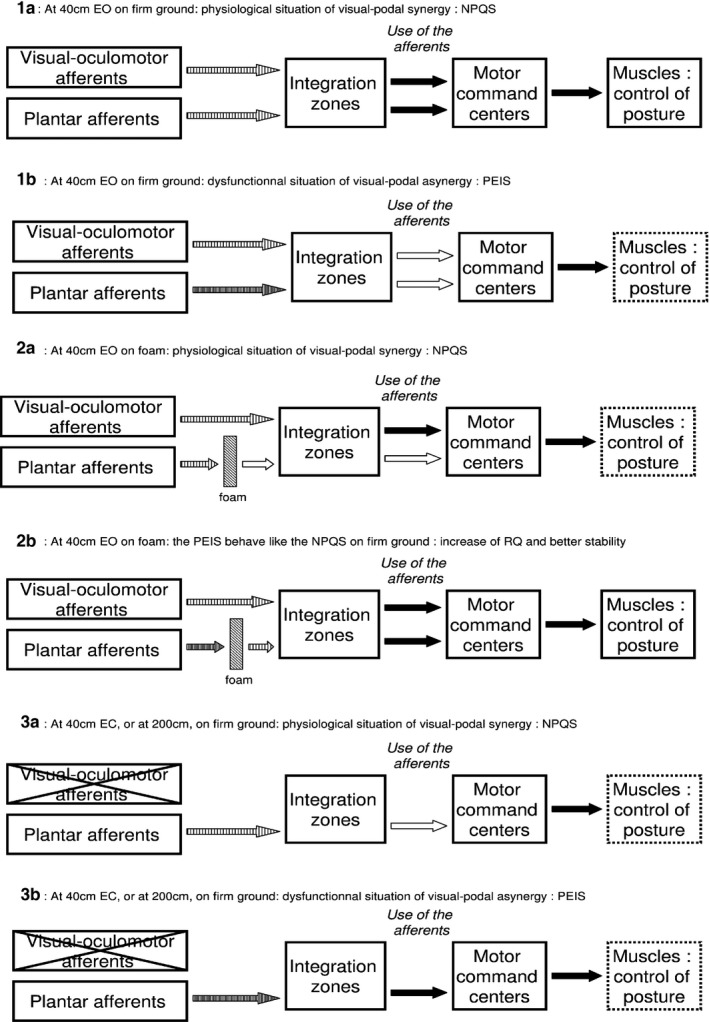
Modelization of visual‐podal synergy/asynergy. At close distance, EO, on firm ground (situation 1), the plantar and visual afferents of the Normal Plantar Quotient Subjects (NPQS) are clear and easily processed by the central nervous system, resulting in an efficient postural control (1a). Among the Plantar Exteroceptive Inefficient Subjects (PEIS) (1b), the increased plantar signal (tight‐hatched arrow) makes it more difficult to process and results in both a decreased Plantar Quotient (PQ) and Romberg Quotient (RQ) (white arrows) and in an impaired postural control (dotted box). Foam decreases the plantar signal (situation 2), resulting in increased body sways among the NPQS (2a) and in a better balanced use of plantar and visual afferents and consecutive better postural control among the PEIS (2b). The decrease in visual and oculomotor afferents (situation 3) reduces the use of plantar cues among the NPQS (decrease in PQ) (3a), and facilitates the integration of the PEIS’ noisy plantar signal (increase in PQ) (3b)

This rationale could also explain the effects that we observe here. In the normal situation of visual‐podal synergy, plantar and visual signals are clear, easily used and equally processed by the CNS, producing an efficient postural control (Figure 5:1a). In contrast, in the dysfunctional situation of PEI, the plantar signal is increased (Foisy & Kapoula, 2016), making it more difficult to process (Weerakkody et al., 2003). As both the PQ and RQ are lower in this case, it suggests that both kinds of afferents are processed in the common integration zones which act as a filter for both these cues at the same time, even if only one of these signals is distorted. It results in a visual‐podal asynergy and in a less efficient postural control (Figure 5:1b).

When foam (Dépron^®^) is placed beneath the feet, it smoothes plantar pressure distribution (Chiang & Wu, 1997; Wu & Chiang, 1997) and decreases the plantar signal (Yi & Park, 2009). This filtering has different effects depending on the subjects. The decrease in the NPQS’ normal feet cutaneous afferents results in an increase in body sway (Figure 5:2a), whereas the decrease in the PEIS’ noisy plantar signal reestablishes the balance between feet and eyes cues in the common integration zones, thus improving the control of posture (Figure 5:2b).

When visual and oculomotor afferents are reduced, that is, EC or at far distance (Le & Kapoula, 2007), the NPQS’ PQ decreases. It shows that among these subjects, the deprivation of these cues hinders the processing of the plantar afferents, suggesting that both kinds of information normally need to be compared in the common integrative zones (Figure 5:3a). On the contrary, the PEIS’ PQ increases in this situation, which suggests that the common zones can focus on the processing of the noisy plantar signal and the use of these afferents (Figure 5:3b).

Dujols (1991) proposed the term “visual‐podal conflict” in order to explain his clinical observation. The word “conflict” usually refers to incongruent information arising from two or more inputs; for example, motion sickness, resulting from conflicting vestibular and visual signals. It is also used for artificial conflicting situations created for the needs of an experiment. For instance, convergent prisms produce incongruent visual and oculomotor messages in the estimation of distance (Kapoula & Le, 2006). Here, given that there is no incongruence of information between eye and feet information, we prefer evoking “visual‐podal synergy” versus “visual‐podal asynergy”, which better reflects our results. This way of thinking is a new hypothetical interpretation that we propose, which is also different from the preceding model of “sensory re‐weighting”. In the latter the “weight” which is attributed to the use of the different signals is dynamically adjusted according to their reliability (Dokka, Kenyon, Keshner, & Kording, 2010; Ernst & Bülthoff, 2004). In our new theoretical scheme, we show that, at close distance, there is a synergy between visual and podal sources of information; if one is not properly functioning the other one is unable to be used either.

These findings could explain the discrepancies of the literature about whether those two inputs act independently or not on postural control: Fransson, Gomez, Patel, and Johansson (2007) did not find any significant combined postural effect of the deprivation of both visual and somaesthetic cues among subjects who were fixating a target at 150 cm in front of their eyes. Those results seems in line with ours (i.e., there is no interaction is eye and feet afferents at far distance). In contrast, Blackburn, Riemann, Myers, and Lephart (2003) found an interaction between those factors, but the distance of the visual target was not specified in their study.

### 4.3 Effects of foam surfaces and Anterior Bar on the PQ, RQ, and postural performances

Firstly, concerning the PQ, among the NPQS it is smaller with Dynachoc^®^ at 40 cm EO and at 200 cm EC. This result, along with the more significant action of Dépron^®^ on PQ modifications following eye closure and increase in distance, suggests that Dépron^®^ suppresses better the effects of PEI than Dynachoc^®^.

As concerns RQ, as mentioned above, the group comparisons results showed that, at 40 cm, the PEIS have a significantly lower RQ than the NPQS on each ground condition, except on Dépron^®^. It confirms that Dépron^®^ is better able to suppress the visual‐podal asynergy than Dynachoc^®^ and AB^®^.

Concerning postural performance, the group comparison results showed that, at 40 cm EO, the PEIS are more unstable than the NPQS on firm ground and have a tendency to be more stable on Dépron^®^. The absence of difference in the two groups with Dynachoc^®^ added to the previously described results, suggests that Dépron^®^ better detects PEI than Dynachoc^®^. It can be explained by the greater thickness of the material (respectively, 6 mm against 3mm) along with a softer composition (respectively, shore rating of 20A and density of 33 kg/m^3^ against shore rating of 35A and density of 350 kg/m^3^). These characteristics can help obtain a better plantar pressure distribution, that is, a more important decrease in the excess of pressure beneath the first metatarsal head, which is considered to cause the PEI (see Foisy & Kapoula, 2016; Janin, 2009). On the whole, those results show that, even with differences that could seem very small, slightly different foam surfaces have statistically different effects on postural control. They confirm and complement the results of Di Berardino et al. (2009), who found significant differences in the postural effects of thicker foam pads (10 or 8 cm thick, density of 25 or 100 kg/m^3^).

Finally, the AB^®^ does not have the same effect on the RQ than the foam surfaces. As mentioned above, the group comparisons’ results showed that, at 40 cm, the PEIS have a significantly lower RQ than the NPQS, except on Dépron^®^, suggesting that the AB^®^ does not suppress the visual‐podal asynergy contrary to Dépron^®^. It may be due to the fact that the plantar insert increases the feet tactile signal (Foisy et al., 2015), while foam lessens it (Yi & Park, 2009). In the situation of visual‐podal asynergy, additional information must be more difficult for the CNS to process than the attenuation of a peripheral noisy source of afferents. However, this stimulation keeps a positive effect on postural control in terms of decrease in Surface area and Variance of Speed, mainly at 200 cm.

## 5 Conclusion

This study underscores that physiologically, eye and feet afferents operate in a synergic way to ensure postural control but this functional synergy is broken among the PEIS; they show a visual‐podal asynergy. It also clarifies the pathophysiology of PEI (see Foisy & Kapoula, 2016): given that its effects are only present at close distance, it suggests that they are a consequence of the visual‐podal asynergy rather than of a physical lesion.

These findings have many clinical implications. Firstly, they confirm that the Surface area PQ and RQ are simple, noninvasive and valuable means to assess interindividual differences and sensorial preferences among young and healthy subjects, in agreement with previous work (Foisy & Kapoula, 2016). Second, it suggests that Dépron^®^ is preferable than Dynachoc^®^ to bring out the effects of PEI. It also shows that, despite its positive action on postural control, the AB^®^ does not suppress the visual‐podal asynergy. Finally, given that PEIS are more unstable and have trouble to integrate both their plantar and visual afferents, it is likely that such a latent dysfunctional situation could evolve toward symptoms in the long run. Further research is required to confirm this assumption. The measure of PQ could thus be used for prevention and follow up. Knowing that Dépron^®^ is not suitable for utilization in foot orthoses, it would also be useful to develop further research in order to identify the best materials able to suppress PEI and visual‐podal asynergy that could be used either in insoles or shoes. It may have clinical implications, for example, in preventing falls among the elderly.

## Conflict of Interest

None of the authors have potential conflicts of interest to be disclosed.
