# Supplementary material for: Plantar Exteroceptive Inefficiency causes an asynergic use of plantar and visual afferents for postural control: Best means of remediation
Source: Brain Behav. 2017 May 1;7(6):e00658. doi: 10.1002/brb3.658 (PMC5474697; doi:10.1002/brb3.658)
Supplement: Supplementary file 1 [file BRB3-7-e00658-s001.doc]

Table S1

*Subject’s characteristics*

| Subject # | Gender | Age | Height  (cm) | Weight  (kg) | TNO | Parinaud  (mean) | Accom-modation | NCP |
| --- | --- | --- | --- | --- | --- | --- | --- | --- |
| 1 | M | 20 | 182 | 63 | 30 | 2 | 10,72 | 5,00 |
| 2 | M | 26 | 176 | 84 | 30 | 2 | 7,59 | 7,00 |
| 3 | M | 27 | 177 | 80 | 30 | 2 | 7,23 | 5,00 |
| 4 | F | 31 | 159 | 56 | 60 | 2,5 | 9,84 | 6,17 |
| 5 | F | 26 | 169 | 58 | 15 | 2 | 6,74 | 7,83 |
| 6 | F | 25 | 163 | 55 | 60 | 2 | 9,52 | 7,00 |
| 7 | F | 23 | 165 | 55 | 30 | 2 | 5,88 | 5,33 |
| 8 | M | 24 | 172 | 64 | 30 | 2 | 9,52 | 4,33 |
| 9 | M | 26 | 177 | 70 | 30 | 2 | 13,04 | 3,83 |
| 10 | M | 33 | 185 | 73 | 60 | 2 | 7,79 | 4,75 |
| 11 | M | 27 | 180 | 84 | 60 | 2 | 12,00 | 6,17 |
| 12 | F | 23 | 171 | 53 | 15 | 2 | 9,68 | 4,83 |
| 13 | M | 26 | 178 | 68 | 20 | 2 | 8,11 | 4,33 |
| 14 | M | 27 | 185 | 73 | 15 | 2 | 13,33 | 4,83 |
| 15 | M | 25 | 166 | 73 | 30 | 2 | 11,54 | 5,33 |
| 16 | M | 22 | 181 | 80 | 60 | 2 | 14,29 | 6,67 |
| 17 | F | 23 | 174 | 60 | 30 | 2 | 9,09 | 7,50 |
| 18 | F | 21 | 160 | 47 | 60 | 2 | 10,17 | 6,67 |
| 19 | M | 25 | 183 | 73 | 30 | 2 | 9,38 | 7,67 |
| 20 | F | 21 | 170 | 55 | 30 | 2 | 8,33 | 8,00 |
| 21 | M | 22 | 179 | 68 | 60 | 2 | 10,17 | 5,50 |
| 22 | M | 26 | 163 | 68 | 30 | 2 | 8,70 | 5,17 |
| 23 | F | 23 | 170 | 56 | 30 | 2 | 9,09 | 6,50 |
| 24 | M | 26 | 175 | 70 | 60 | 2 | 7,79 | 4,50 |
| 25 | M | 28 | 175 | 68 | 30 | 2 | 9,38 | 6,83 |
| 26 | M | 24 | 178 | 75 | 60 | 2 | 8,57 | 4,00 |
| 27 | F | 31 | 168 | 59 | 30 | 2 | 6,67 | 4,67 |
| 28 | F | 25 | 157 | 59 | 30 | 2 | 10,17 | 2,83 |
| 29 | F | 21 | 169 | 61 | 60 | 2 | 9,38 | 8,17 |
| 30 | F | 24 | 176 | 70 | 60 | 2 | 13,04 | 1,00 |
| 31 | F | 22 | 165 | 62 | 60 | 2 | 8,33 | 1,00 |
| 32 | M | 24 | 177 | 95 | 30 | 2 | 10,71 | 4,17 |
| 33 | F | 31 | 160 | 50 | 60 | 2 | 6,32 | 6,33 |
| 34 | M | 24 | 176 | 69 | 30 | 2 | 8,22 | 5,00 |
| 35 | F | 22 | 147 | 53 | 30 | 2 | 8,96 | 2,00 |
| 36 | F | 21 | 162 | 55 | 60 | 2 | 9,38 | 4,00 |
| 37 | F | 28 | 166 | 60 | 60 | 2 | 9,09 | 3,67 |
| 38 | F | 25 | 165 | 53 | 60 | 2 | 11,32 | 4,83 |
| 39 | F | 22 | 153 | 54 | 60 | 2 | 9,09 | 2,33 |
| 40 | F | 22 | 170 | 65 | 60 | 2 | 7,41 | 2,83 |
| 41 | F | 33 | 169 | 63 | 30 | 2 | 6,32 | 5,50 |
| 42 | F | 23 | 162 | 55 | 15 | 2 | 10,00 | 7,17 |
| 43 | M | 32 | 165 | 65 | 60 | 2 | 9,68 | 3,00 |
| 44 | M | 24 | 180 | 70 | 60 | 3 | 8,82 | 5,50 |
| 45 | F | 22 | 163 | 52 | 15 | 2 | 8,45 | 3,00 |
| 46 | F | 21 | 164 | 43 | 30 | 2 | 10,53 | 2,00 |
| 47 | F | 25 | 162 | 58 | 60 | 2 | 10,17 | 6,33 |
| 48 | F | 27 | 175 | 58 | 60 | 2 | 10,00 | 6,67 |
| *M* |  | 25,0 | 170,1 | 63,7 | 41,98 | 2,03 | 9,37 | 5,06 |
| *SD* |  | 3,3 | 8,6 | 10,5 | 17,34 | 0,16 | 1,85 | 1,82 |

*Note*. For each subject: stereoacuity (TNO) and visual acuity at close distance (Parinaud, mean of both eyes). Normal values are < 100 for TNO, < 3 for Parinaud and 9.5 ± 2 for the amplitude of accommodation.
